# Phase II Trial of Opaganib Addition in Metastatic Castration‐Resistant Prostate Cancer After Disease Progression on Abiraterone or Enzalutamide

**DOI:** 10.1002/cam4.71633

**Published:** 2026-04-14

**Authors:** Jacqueline T. Brown, Bassel Nazha, Anna C. Ferreira, Kent Armeson, Elizabeth G. Hill, Besim Ogretmen, Shikhar Mehrotra, Alan Brisendine, George Magrath, Terry F. Plasse, Theodore Stewart Gourdin, Omer Kucuk, Michael Lilly

**Affiliations:** ^1^ Department of Hematology and Medical Oncology Emory University School of Medicine Atlanta Georgia USA; ^2^ Winship Cancer Institute of Emory University Atlanta Georgia USA; ^3^ Department of Public Health Sciences Medical University of South Carolina Charleston South Carolina USA; ^4^ Hollings Cancer Center Medical University of South Carolina Charleston South Carolina USA; ^5^ Department of Opthalmology Medical University of South Carolina Charleston South Carolina USA; ^6^ RedHill Biopharma Ltd Tel Aviv Israel; ^7^ Division of Hematology‐Oncology, Department of Medicine Medical University of South Carolina Charleston South Carolina USA

**Keywords:** abiraterone, enzalutamide, opaganib, prostate cancer, sphingolipid

## Abstract

**Introduction:**

Opaganib is a first‐in‐class oral sphingolipid metabolism inhibitor that inhibits sphingosinekinase 2 (SphK2) and dihydroceramide desaturase (DES) and that has a demonstrated safety and preliminary anti‐cancer activity signal in a Phase I study.

**Methods:**

In this phase II trial, patients with metastatic castration‐resistant prostate cancer who had disease progression on novel hormonal agents (NHAs) abiraterone or enzalutamide were enrolled and treated with opaganib while continuing their NHA. After safety lead‐in cohorts, the trial enrolled cohort 2 (abiraterone + opaganib 500 mg Q 12 h) and cohort 3 (enzalutamide + opaganib 500 mg Q 12 h). The primary efficacy endpoint was the proportion of patients with disease control at Day 113. The postulated disease control rate was 10%. Secondary efficacy endpoints include prostate‐specific antigen (PSA) progression‐free survival (PSA‐PFS) and PSA response rates. The primary safety endpoint was the incidence of adverse events (AEs).

**Results:**

The disease control rates were 15% (95% CI = 4%–35%, 4 of 26 patients) in cohort 2 and 9% (95% CI = 2%–24%, 3 of 34 patients) in cohort 3. The median PSA‐PFS was 56 days (95% CI = 35–112 days) in cohort 2 and 55 days (95% CI = 35–56 days) in cohort 3. The most common AEs of grade 3 or higher were hypertension (8%) and musculoskeletal AEs (8%) in cohort 2 and grade 3 anemia (18%) in cohort 3.

**Conclusion:**

The trial did not meet its primary objective of demonstrating 30% disease control at 113 days. However, subjects who experienced a PSA response or stabilization warrant further exploration for biomarkers of response.

**Trial Registration:**

ClinicalTrials.gov number: NCT04207255

## Graphical abstract

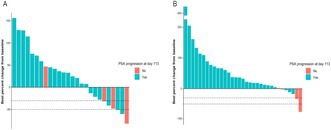

## 1 Introduction

Although metastatic castration‐resistant prostate cancer (mCRPC) is incurable, therapeutic developments over the last two decades have significantly improved outcomes in affected patients. Taxane chemotherapeutics [1, 2, 3], novel hormonal agents (NHA) including second‐generation androgen receptor (AR) and androgen biosynthesis inhibitors like enzalutamide and abiraterone, respectively [4, 5, 6], and most recently, the prostate‐specific membrane antigen‐targeted radioligand lutetium (Lu‐177) vipivotide tetraxetan all offer survival benefit [4, 5]. However, average survival after the development of castration resistance is < 3 years, and prostate cancer remains the second leading cause of cancer‐related death in the US for men [6]. For these reasons, novel therapeutic alternatives are desperately needed and well‐tolerated oral agents are attractive from a quality‐of‐life standpoint.

Sphingomyelin is an essential component of cellular membranes and is converted by sphingomyelinase to ceramide. Ceramidase subsequently converts ceramide to sphingosine, a substrate phosphorylated by sphingosine kinases to produce sphingosine‐1‐phosphate (S1P) [7]. Ceramide and sphingosine have pro‐apoptotic properties that affect tumors but not normal cells, whereas S1P is associated with cell proliferation, angiogenesis, and anti‐apoptosis [8]. The metabolic balance of ceramide and S1P has been denoted as the “sphingolipid rheostat,” and its manipulation by malignant cells toward increased S1P generation by the sphingosinekinases SphK1 or SphK2 has led to the identification of selective kinase inhibitors capable of tumor suppression [9].

Opaganib is a first‐in‐class, oral, selective inhibitor of sphingosine kinase 2 (SphK2) over SphK1, enzymes that convert sphingosine to S1P. Pre‐clinical work suggested SphK2 as a promising anticancer target in various cancer types [10, 11]. In addition to tilting the rheostat toward pro‐apoptotic ceramide by decreasing S1P levels, opaganib has been shown to inhibit dihydroceramide desaturase (DES) activity, leading to dihydroceramide accumulation, as well as attenuation of key proliferative signaling pathways involving Ras/Raf/MRK/ERK and Ras/PI3K/AKT [11]. It also targets the oncogenic c‐MYC for proteasomal degradation, inhibits TNF‐a‐driven inflammation and NFκB activation, and stimulates tumor autophagy [12, 13, 14, 15]. Of particular interest in prostate cancer is the ability of opaganib to reduce AR expression in prostate cancer cells, thus altering a primary resistance mechanism that arises in response to treatment with androgen deprivation and NHA [16, 17, 18]. Further, opaganib's oral formulation could address the patient‐centered need to prolong the chemotherapy‐free period in CRPC [19].

The first‐in‐human phase I study of opaganib across solid tumor types demonstrated the drug's safety and preliminary signal of anti‐cancer activity [20]. In this phase II trial, patients with mCRPC who progressed while receiving enzalutamide or abiraterone were enrolled and treated with opaganib while continuing their original NHA. The trial's primary objective was the assessment of disease control, defined as stable disease or better after 113 days (16 weeks) of treatment and measured by a composite metric incorporating PSA and imaging criteria.

## 2 Methods

### 2.1 Study Population

The study enrolled patients with mCRPC who had been treated with at least one oral androgen signaling blocker (abiraterone or enzalutamide) and no prior chemotherapy for castration‐resistant disease. Patients who had disease progression on abiraterone or enzalutamide were able to enroll, with the addition of opaganib while continuing their NHA. The study sites included the Medical University of South Carolina (MUSC) (Charleston, South Carolina, USA) and Emory University (Atlanta, GA, USA). The trial enrolled patients between March 20, 2020 and December 29, 2022. All patients provided written informed consent, and the study had institutional review board approval (IRB) at the two study sites (MUSC IRB and Emory IRB). The study was registered at ClinicalTrials.gov (NCT04207255). The full list of inclusion and exclusion criteria appears in the Supporting Information S1: Section of the manuscript.

### 2.2 Trial Design

The trial was a phase II non‐randomized efficacy trial of patients with mCRPC. The trial included a brief safety lead‐in in cohorts 1a (abiraterone 1000 mg daily + opaganib 250 mg every 12 h, 3 subjects) and 1b (enzalutamide 160 mg daily + opaganib 250 mg every 12 h, 3 subjects). Once both cohorts 1a and 1b were completed without dose‐limiting toxicities, the trial proceeded with enrollment into the phase II component, with cohort 2 (abiraterone 1000 mg daily + opaganib 500 mg every 12 h, subjects previously on abiraterone) and cohort 3 (enzalutamide 160 mg daily + opaganib 500 mg every 12 h, subjects previously on enzalutamide). Both cohorts 2 and 3 used the same Simon two‐stage design testing the null hypothesis that the disease control rate was ≤ 10% versus the alternative that the disease control rate was ≥ 30%. The null hypothesis of disease control rate ≤ 10% is based on overall efficacy of second‐line androgen receptor pathway inhibitor NHA of about 10% after progression on first‐line therapies with NHA [21]. Out of 20 patients enrolled in stage one, if at most 1 patient achieved disease control at 16 weeks, the cohort closed to accrual. Otherwise, an additional seven patients were enrolled for a total sample size in each cohort of *N* = 27, and the null hypothesis was rejected if at least six patients achieved disease control. Neither cohort paused enrollment for evaluation of futility. This design controlled the type I error rate at 5% with 86% power, and a probability of early termination under the null hypothesis of 39%. A protocol amendment approved enrollment to exceed 27 for the purposes of PK sampling, provided the cohort proceeded past the stage one futility analysis. Subjects continued treatment until the development of progressive disease, intolerable toxicity (grade 3 or 4 toxicity that did not resolve to grade 1 or less by 28 days), withdrawal of consent, or investigator decision that continuation was not in the patient's best interest. Each cycle consisted of 28 days (4 weeks). Response was evaluated after 4 cycles (16 weeks). Safety and tolerability were monitored at every study visit.

### 2.3 Primary and Secondary Endpoints

The primary efficacy endpoint was the proportion of patients with disease control during opaganib (plus abiraterone or enzalutamide) therapy, using a composite metric based on prostate‐specific antigen (PSA), RECIST 1.1 [22] soft tissue lesion progression evaluation, and bone lesion progression per Prostate Cancer Working Group 3 (PCWG3) criteria [23]. Specifically, PSA progression was defined as ≥ 25% and ≥ 2 ng/mL increase measured either from baseline (if no decline in PSA from baseline) or nadir (if decline in PSA after baseline), ignoring rises in PSA in the first 12 weeks of treatment. Confirmation of PSA progression was required at least 3 weeks later for patients with PSA decline from baseline. Radiographic soft tissue lesion progression was evaluated only for patients with measurable disease at baseline. Bone progression was defined using the PCWG3 2 + 2 criteria (at least two new lesions on the first post‐treatment scan followed by at least two new lesions on the next bone scan). Disease control after 113 days (16 weeks) of treatment was defined as no PSA progression, stable disease or better (for patients with soft tissue measurable disease at baseline), and no bone progression. Patients classified as having progressed by Day 113 for any of the three metrics (PSA, soft tissue lesion, or bone lesion) were considered not to have met criteria for disease control. The full analysis set (FAS) included all eligible patients who took any study drug and was used for evaluation of the primary endpoint. Patients in the FAS missing PSA or radiographic (soft tissue or bone) evaluation for any reason, thereby precluding assessment of the primary endpoint, were considered not to have met criteria for disease control.

Secondary efficacy endpoints included PSA progression‐free survival (PSA‐PFS) and PSA response rate (PSA50 and PSA30). PSA‐PFS was defined as the time from start of treatment to an increase in PSA ≥ 25% and ≥ 2 ng/mL relative to baseline (if no decline in PSA from baseline) or nadir (if decline in PSA after baseline). For PSA‐PFS, early rises in PSA in the first 12 weeks were included. Additionally, for patients with initial PSA decline, time to PSA progression was censored at the last date of evaluation if PSA progression was unconfirmed. PSA50 was defined as the percentage of participants with an improvement of ≥ 50% in the PSA concentration compared to baseline [23], and PSA30 defined as the percentage of participants with an improvement of ≥ 30% in the PSA concentration compared to baseline [24, 25, 26]. The safety endpoint was the incidence of toxicities as defined by CTCAE criteria, version 5.

### 2.4 Statistical Considerations

For Day 113 disease control, we report proportions and exact binomial 95% confidence intervals (CIs) for cohorts 2 and 3. If a cohort was not closed for futility, efficacy was evaluated based on a one‐sided exact binomial test with *α* = 0.05. Kaplan–Meier estimates of PSA‐PFS were constructed for each cohort. Median time to PSA progression was estimated, and the corresponding 95% confidence intervals were constructed using Greenwood's variance estimate. Waterfall plots were constructed showing patients' best percent change in PSA from baseline, where best percent change was defined as the maximum percent reduction in PSA from baseline or, in the absence of a PSA decline, the minimum percent increase in PSA from baseline. Waterfall plots showing maximum percent change from baseline were also constructed. The safety analysis was conducted using descriptive statistics of the incidence of adverse events (AEs) and serious adverse events (SAEs), all events of death, and any study‐specific issue of concern.

## 3 Results

Overall, the trial enrolled 66 subjects with a median age of 71 years. The baseline clinical and demographic characteristics are shown in Table 1 for the overall study population along with the individual cohorts: cohort 1a (opaganib 250 mg + abiraterone, *N* = 3), cohort 1b (opaganib 250 mg + enzalutamide, *N* = 3), cohort 2 (opaganib 500 mg + abiraterone, *N* = 26), and cohort 3 (opaganib 500 mg + enzalutamide, *N* = 34). Baseline characteristics were generally similar in cohorts 2 and 3, except for a higher prevalence of visceral metastasis in cohort 3 (8/34 = 24% in cohort 3 versus 1/26 = 4% in cohort 2). All but two patients (for whom pathology was unavailable) had adenocarcinomas as a histological diagnosis. No patients received triplet therapy of docetaxel + NHA + ADT in the castration‐sensitive setting (combination was not approved when the trial occurred). Also, no patients had received prior chemotherapy in the castration‐resistant setting.

**TABLE 1:** Baseline clinical and demographic characteristics of enrolled patients.

	Cohort 1a	Cohort 1b	Cohort 2	Cohort 3	Overall (*N* = 66)
	Opaganib 250 mg + Abiraterone (*N* = 3)	Opaganib 250 mg + Enzalutamide (*N* = 3)	Opaganib 500 mg + Abiraterone (*N* = 26)	Opaganib 500 mg + Enzalutamide (*N* = 34)	
Age					
Mean (SD)	67 (2.5)	71 (2.9)	72 (8.0)	71 (7.7)	71 (7.5)
Median [Min, Max]	67 [65, 70]	73 [68, 73]	72 [55, 87]	71.5 [58, 88]	71 [55, 88]
Race					
Asian	0 (0%)	0 (0%)	0 (0%)	1 (3%)	1 (2%)
Black/AA	0 (0%)	0 (0%)	9 (35%)	8 (24%)	17 (26%)
White	3 (100%)	3 (100%)	17 (65%)	25 (74%)	48 (73%)
Baseline PSA					
Mean (SD)	16.3 (14.1)	65.1 (31.1)	21.2 (39.6)	42.8 (82.7)	34.1 (65.4)
Median [min, max]	21.6 [0.2, 27]	53.7 [41.2, 100.3]	9.9 [0.6, 179.5]	10.7 [0.3, 431]	10.6 [0.2, 431]
Gleason score					
6	1 (33)	1 (33)	0 (0)	1 (3)	3 (5)
7	1 (33)	0 (0)	7 (30)	10 (33)	18 (31)
8	0 (0)	0 (0)	9 (39)	6 (20)	15 (25)
9	1 (33)	2 (67)	6 (26)	10 (33)	19 (32)
10	0 (0)	0 (0)	1 (4)	3 (10)	4 (7)
Missing	0	0	3	4	7
Visceral metastases	1 (33%)	0 (0%)	1 (4%)	8 (24%)	10 (15%)
Bone metastases	3 (100%)	3 (100%)	21 (81%)	28 (82%)	55 (83%)
Lymph node involvement	0 (0%)	3 (100%)	8 (31%)	18 (53%)	29 (44%)
Other metastases	0 (0%)	0 (0%)	0 (0%)	1 (3%)	1 (2%)

### 3.1 Efficacy

#### 3.1.1 Disease Control Rate

At the interim futility analysis, three of the first 20 patients in cohort 2 and two of the first 20 patients in cohort 3 had achieved disease control by Day 113. Therefore, both cohorts enrolled patients in stage 2 for a total sample size of *N* = 26 and *N* = 34 trial participants evaluable for the primary endpoint in cohorts 2 and 3, respectively. The disease control rate among cohort 2 patients receiving concurrent abiraterone was 4/26 (15%, 95% CI = 4%–35%; *p* = 0.26). Among cohort 3 patients receiving concurrent enzalutamide, the disease control rate was 3/34 (9%, 95% CI = 2%–24%; *p* > 0.99).

#### 3.1.2 PSA Progression‐Free Survival

**FIGURE 1:**
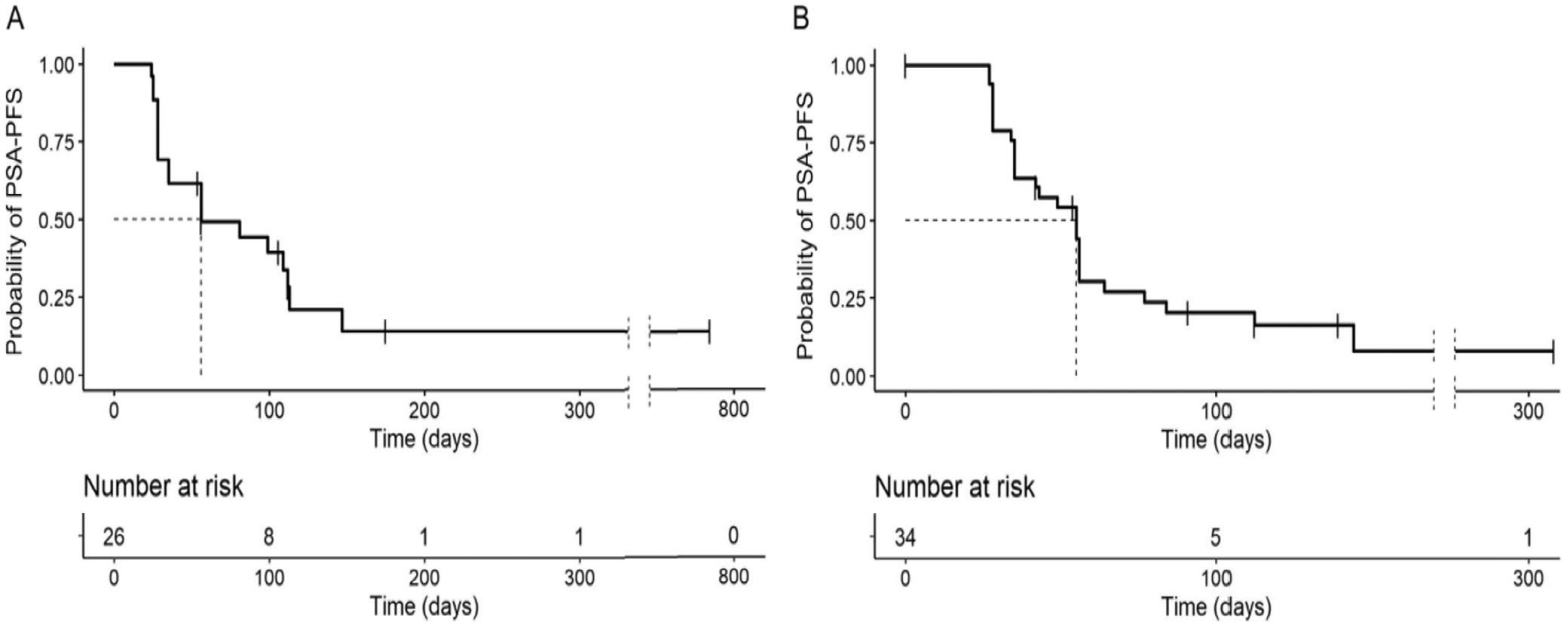
Kaplan–Meier estimates of PSA‐progression‐free (PSA‐PFS) survival in patients receiving opaganib 500 mg and (A) abiraterone (cohort 2) and (B) enzalutamide (cohort 3). PSA‐PFS was defined to include early rises (< 12 weeks) in PSA and required confirmation. For patients with initial PSA decline, time to PSA progression was censored at the last date of evaluation if PSA progression was unconfirmed.

The median PSA progression‐free survival (mPSA‐PFS) in cohort 2 (opaganib 500 mg + abiraterone) was 56 days (95% CI = 35–112 days) as depicted in Figure 1A. The mPSA‐PFS in cohort 3 (opaganib 500 mg + enzalutamide) was 55 days (95% CI = 35–56 days), seen in Figure 1B. A sensitivity analysis (not shown) in which unconfirmed PSA progressions were treated as events rather than censored resulted in similar inference.

#### 3.1.3 PSA Response Rate

**FIGURE 2:**
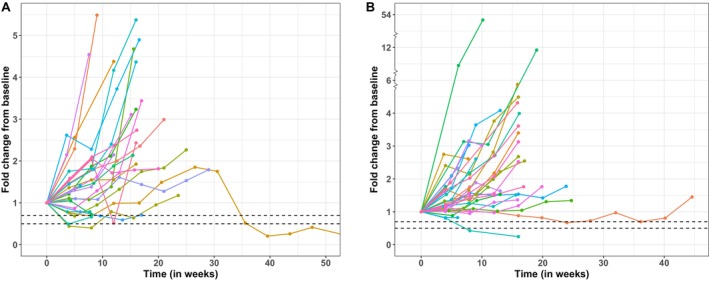
PSA fold‐change (FC) relative to baseline over follow‐up time in patients receiving opaganib 500 mg and (A) abiraterone (cohort 2) or (B) enzalutamide (cohort 3). Dashed lines indicate a PSA reduction of 30% (PSA30; FC = 0.7) and 50% (PSA50; FC = 0.5).

**FIGURE 3:**
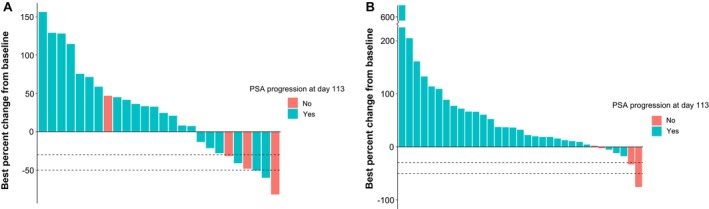
Waterfall plots of best percent change in PSA from baseline in patients received opaganib 500 mg and (A) abiraterone (cohort 2) and (B) enzalutamide (cohort 3), where best percent change is defined as the maximum percent reduction in PSA from baseline or, in the absence of a PSA decline, the minimum percent increase in PSA from baseline. Bars are color‐coded according to PSA progression status at Day 113 based on PCWG3 definition. Dashed lines indicate a PSA reduction of 30% (PSA30) and 50% (PSA50).

The number of patients who had a PSA decline of at least 30% compared to baseline (PSA30) at any time on active treatment was 6 of 26 patients (23%, 95% CI = 9%–44%) in cohort 2 (abiraterone) and 2 of 34 patients (6%, 95% CI = 1%–20%) in cohort 3 (enzalutamide). PSA decline of at least 50% compared to baseline (PSA50) was noted in 3 of 26 patients (12%, 95% CI = 2%–30%) in cohort 2 and 1 of 34 patients (3%, 95% CI = 0%–15%) in cohort 3. PSA fold change relative to baseline over time in patients receiving opaganib plus abiraterone or enzalutamide is depicted in Figure 2A,B, respectively. The best percent change in PSA from baseline in these same cohorts is shown in Figure 3A,B, respectively. Three of the 6 patients (50%) in cohort 2 who achieved a PSA reduction by at least 30%, and both patients (100%) in cohort 3 who achieved a PSA reduction by at least 30% remained progression‐free by PSA measurement at Day 113.

### 3.2 Safety

In the trial's overall study population, 80% (53/66) had at least 1 AE of any grade, and 26% (17/66) experienced grade 3 or higher AEs. All AEs per cohort, regardless of attribution (treatment‐related or not), are presented in Table S1. Neither cohort 1a (*N* = 3) nor cohort 1b (*N* = 3) had grade 2, 3, or 4 AEs. Common to both cohorts was grade 1 fatigue (1 in cohort 1a and 2 in cohort 1b).

In cohort 2 (*N* = 26), most AEs were grade 1 or 2. The most common grade 1 AEs were nervous system AEs (38%), gastrointestinal AEs (35%), and fatigue (27%). Four subjects (15%) had grade 2 nervous system AEs. In cohort 2, there was an 8% incidence of grade 3 hypertension and an 8% incidence of grade 3 musculoskeletal AEs with no grade 4 AEs. Notably, 1 patient had a grade 5 ventricular arrhythmia AE (felt by the treating investigators to be unrelated to the study drug) (Table 2). Grade 1 AEs in cohort 3 (*N* = 34) included a predominance of abdominal AEs (41%) along with musculoskeletal (21%) and nervous system AEs (18%). Grade 2 nervous system AEs were also seen in 12% of subjects. The most common grade 3 AE was anemia, affecting 6 subjects (18%). Cohort 3 had noticeably more grade 4 adverse events; these included sepsis in 2 subjects (6%) and cerebral edema in 1 (3%), in addition to one grade 5 myocardial infarction (felt by the treating investigators to be unrelated to the study drug) (Table 2).

**TABLE 2:** Frequency of all adverse events by grade within system organ class (SOC) for cohorts 2 and 3.

SOC, *n* (%) Adverse event, *n* (%)	Cohort 2					Cohort 3				
	(500 mg opaganib + A) *N* = 26					(500 mg opaganib + E) *N* = 34				
	Grade					Grade				
	1	2	3	4	5	1	2	3	4	5
**Blood**	1 (4)	1 (4)	—	—	—	—	—	6 (18)	—	—
Anemia	1 (4)	1 (4)	—	—	—	—	—	6 (18)	—	—
Leukocytosis	1 (4)	—	—	—	—	—	—	1 (3)	—	—
**Cardiac**	—	—	—	—	1 (4)	1 (3)	—	1 (3)	—	1 (3)
Myocardial infarction	—	—	—	—	—	—	—	—	—	1 (3)
Palpitations	—	—	—	—	—	1 (3)	—	—	—	—
Sinus tachycardia	—	—	—	—	—	—	—	1 (3)	—	—
Ventricular arrhythmia	—	—	—	—	1 (4)	—	—	—	—	—
**Ear**	2 (8)	—	—	—	—	1 (3)	—	—	—	—
Tinnitus	1 (4)	—	—	—	—	1 (3)	—	—	—	—
Vertigo	1 (4)	—	—	—	—	—	—	—	—	—
**Eye**	3 (12)	1 (4)	—	—	—	3 (9)	—	—	—	—
Blurred vision	1 (4)	1 (4)	—	—	—	1 (3)	—	—	—	—
Flashing lights	2 (8)	—	—	—	—	1 (3)	—	—	—	—
Floaters	—	—	—	—	—	1 (3)	—	—	—	—
**Gastrointestinal**	9 (35)	3 (12)	1 (4)	—	—	14 (41)	1 (3)	—	—	—
Abdominal distension	—	—	—	—	—	1 (3)	—	—	—	—
Abdominal pain	1 (4)	—	1 (4)	—	—	4 (12)	—	—	—	—
Bloating	—	—	—	—	—	1 (3)	—	—	—	—
Constipation	2 (8)	1 (4)	—	—	—	4 (12)	—	—	—	—
Diarrhea	4 (15)	—	—	—	—	2 (6)	—	—	—	—
Dry mouth	1 (4)	—	—	—	—	2 (6)	—	—	—	—
Dyspepsia	1 (4)	—	—	—	—	1 (3)	—	—	—	—
Dysphagia	—	1 (4)	—	—	—	1 (3)	—	—	—	—
Fecal incontinence	—	1 (4)	—	—	—	—	1 (3)	—	—	—
Flatulence	—	—	—	—	—	1 (3)	—	—	—	—
Gastroesophageal reflux disease	1 (4)	—	—	—	—	1 (3)	—	—	—	—
Nausea	5 (19)	—	—	—	—	5 (15)	—	—	—	—
Oral dysesthesia	—	1 (4)	—	—	—	—	—	—	—	—
Vomiting	1 (4)	—	—	—	—	2 (6)	—	—	—	—
**General**	7 (27)	3 (12)	2 (8)	—	—	10 (29)	4 (12)	—	—	—
Edema limbs	—	1 (4)	—	—	—	2 (6)	—	—	—	—
Fatigue	7 (27)	2 (8)	1 (4)	—	—	6 (18)	2 (6)	—	—	—
Fever	1 (4)	—	—	—	—	1 (3)	2 (6)	—	—	—
Gait disturbance	1 (4)	—	—	—	—	—	—	—	—	—
Injection site reaction	1 (4)	—	—	—	—	—	—	—	—	—
Localized edema	1 (4)	—	—	—	—	—	—	—	—	—
Malaise	—	—	—	—	—	1 (3)	—	—	—	—
Pain	—	—	1 (4)	—	—	3 (9)	—	—	—	—
**Infections**	3 (12)	1 (4)	—	—	—	1 (3)	—	—	2 (6)	—
Catheter related infection	—	—	—	—	—	1 (3)	—	—	—	—
Sepsis	—	1 (4)	—	—	—	—	—	—	2 (6)	—
Sinusitis	1 (4)	—	—	—	—	—	—	—	—	—
Tooth infection	—	—	—	—	—	1 (3)	—	—	—	—
Upper respiratory infection	2 (8)	—	—	—	—	1 (3)	—	—	—	—
Urinary tract infection	—	1 (4)	—	—	—	—	—	1 (3)	—	—
**Injury**	2 (8)	1 (4)	—	—	—	2 (6)	—	—	—	—
Bruising	2 (8)	—	—	—	—	—	—	—	—	—
Fall	—	1 (4)	—	—	—	1 (3)	—	—	—	—
Fracture	—	—	—	—	—	1 (3)	—	—	—	—
**Investigations**	2 (8)	1 (4)	1 (4)	—	—	1 (3)	2 (6)	2 (6)	1 (3)	—
Activated partial thromboplastin time prolonged	—	—	—	—	—	1 (3)	2 (6)	—	—	—
Alkaline phosphatase increased	—	—	—	—	—	—	—	1 (3)	—	—
Blood bilirubin increased	—	—	—	—	—	—	—	1 (3)	—	—
Creatinine increased	—	1 (4)	1 (4)	—	—	—	—	—	—	—
Lymphocyte count decreased	—	—	—	—	—	—	—	1 (3)	—	—
Platelet count decreased	—	—	—	—	—	—	—	—	1 (3)	—
Weight gain	1 (4)	—	—	—	—	—	—	—	—	—
Weight loss	1 (4)	—	—	—	—	—	—	—	—	—
**Metabolism**	4 (15)	—	—	—	—	2 (6)	2 (6)	1 (3)	1 (3)	—
Anorexia	3 (12)	—	—	—	—	4 (12)	—	—	—	—
Dehydration	—	—	—	—	—	—	2 (6)	1 (3)	—	—
Hypokalemia	2 (8)	—	—	—	—	—	—	—	—	—
Hypomagnesemia	1 (4)	—	—	—	—	—	—	—	—	—
Hyponatremia	—	—	—	—	—	—	—	—	1 (3)	—
**Musculoskeletal**	4 (15)	2 (8)	2 (8)	—	—	7 (21)	2 (6)	2 (6)	—	—
Arthralgia	—	—	—	—	—	2 (6)	—	—	—	—
Arthritis	—	1 (4)	—	—	—	—	—	—	—	—
Back pain	2 (8)	—	1 (4)	—	—	2 (6)	—	2 (6)	—	—
Bone pain	2 (8)	—	—	—	—	2 (6)	—	—	—	—
Chest wall pain	—	—	—	—	—	1 (3)	—	—	—	—
Flank pain	—	—	1 (4)	—	—	1 (3)	1 (3)	—	—	—
Generalized muscle weakness	1 (4)	—	—	—	—	1 (3)	1 (3)	—	—	—
Muscle cramp	1 (4)	—	—	—	—	—	—	—	—	—
Muscle weakness lower limb	2 (8)	—	—	—	—	—	—	—	—	—
Muscle weakness right sided	—	—	—	—	—	1 (3)	—	—	—	—
Neck pain	1 (4)	—	—	—	—	—	—	1 (3)	—	—
Pain in extremity	—	2 (8)	—	—	—	1 (3)	—	1 (3)	—	—
**Nervous**	10 (38)	4 (15)	1 (4)	—	—	6 (18)	4 (12)	—	1 (3)	—
Ataxia	1 (4)	—	—	—	—	—	—	—	—	—
Dizzinescol9" align="center">—	1 (3)	—
Ataxia	1 (4)	—	—	—	—	—	—	—	—	—
Dizziness	4 (15)	2 (8)	—	—	—	5 (15)	—	—	—	—
Dysarthria	1 (4)	—	—	—	—	—	—	—	—	—
Dysgeusia	2 (8)	1 (4)	—	—	—	—	1 (3)	—	—	—
Edema cerebral	—	—	—	—	—	—	—	—	1 (3)	—
Headache	5 (19)	—	—	—	—	1 (3)	—	—	—	—
Intracranial hemorrhage	—	—	—	—	—	—	1 (3)	—	—	—
Lethargy	—	—	—	—	—	—	1 (3)	—	—	—
Memory impairment	1 (4)	1 (4)	—	—	—	—	—	—	—	—
Paresthesia	—	—	—	—	—	1 (3)	—	—	—	—
Peripheral motor neuropathy	—	—	—	—	—	—	1 (3)	—	—	—
Peripheral sensory neuropathy	—	—	—	—	—	2 (6)	—	—	—	—
Seizure	—	—	—	—	—	—	1 (3)	—	—	—
Stroke	—	—	1 (4)	—	—	—	—	—	—	—
Transient ischemic attacks	1 (4)	—	—	—	—	—	—	—	—	—
Tremor	1 (4)	—	—	—	—	—	—	—	—	—
**Psychiatric**	3 (12)	1 (4)	—	—	—	1 (3)	3 (9)	—	—	—
Anxiety	—	—	—	—	—	—	1 (3)	—	—	—
Confusion	1 (4)	—	—	—	—	2 (6)	2 (6)	—	—	—
Hallucinations	2 (8)	1 (4)	—	—	—	1 (3)	—	—	—	—
Restlessness	1 (4)	—	—	—	—	—	—	—	—	—
**Renal**	4 (15)	2 (8)	—	—	—	2 (6)	2 (6)	—	—	—
Bladder spasm	1 (4)	—	—	—	—	—	—	—	—	—
Hematuria	1 (4)	1 (4)	—	—	—	1 (3)	—	—	—	—
Proteinuria	—	1 (4)	—	—	—	—	1 (3)	—	—	—
Urinary frequency	1 (4)	—	—	—	—	—	—	—	—	—
Urinary incontinence	1 (4)	—	—	—	—	1 (3)	1 (3)	—	—	—
Urinary tract obstruction	—	—	—	—	—	1 (3)	1 (3)	—	—	—
Urinary tract pain	1 (4)	—	—	—	—	—	—	—	—	—
**Reproductive**	1 (4)	1 (4)	—	—	—	1 (3)	—	—	—	—
Pelvic pain	1 (4)	1 (4)	—	—	—	1 (3)	—	—	—	—
**Respiratory**	1 (4)	—	—	—	—	2 (6)	—	1 (3)	—	—
Dyspnea	—	—	—	—	—	3 (9)	—	—	—	—
Pleural effusion	—	—	—	—	—	1 (3)	—	—	—	—
Pneumonitis	—	—	—	—	—	—	—	1 (3)	—	—
Sinus disorder	1 (4)	—	—	—	—	—	—	—	—	—
**Skin**	1 (4)	—	—	—	—	4 (12)	—	—	—	—
Hyperhidrosis	1 (4)	—	—	—	—	—	—	—	—	—
Pruritus	—	—	—	—	—	1 (3)	—	—	—	—
Rash acneiform	—	—	—	—	—	3 (9)	—	—	—	—
**Vascular**	3 (12)	2 (8)	2 (8)	—	—	2 (6)	2 (6)	—	—	—
Flushing	1 (4)	—	—	—	—	—	—	—	—	—
Hematoma	—	—	—	—	—	—	1 (3)	—	—	—
Hot flashes	2 (8)	—	—	—	—	2 (6)	—	—	—	—
Hypertension	1 (4)	1 (4)	2 (8)	—	—	—	1 (3)	—	—	—
Hypotension	—	—	—	—	—	1 (3)	—	—	—	—
Thromboembolic event	—	1 (4)	—	—	—	—	—	—	—	—

**FIGURE 4:**
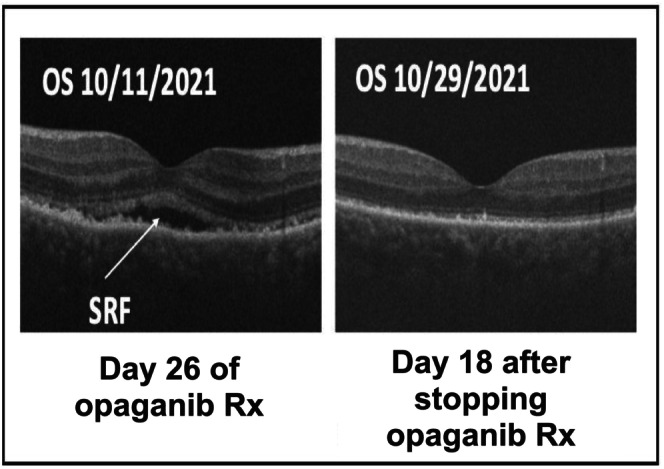
Optical coherence tomography of left eye in patient 103,193–2027. SRF, subretinal fluid; Rx, therapy.

Rare adverse events of special interest were uncommon neuropsychiatric symptoms such as visual and auditory hallucinations and chromatic aberrations. Their presence in our subjects was anticipated based on findings in the first‐in‐human trial [20]. These visual changes were described as colored spots on sheets or toilet water, light bulbs being green or blue, sparklers seen when the eyes were closed, or a sensation of being amidst a glowing white fireworks display. Visual and auditory hallucinations were also reported. Two patients reported the triggering of visual hallucinations with the co‐consumption of alcohol or cannabidiol. A patient who experienced visual hallucinations also reported a new central visual field defect in the left eye. At an ophthalmology evaluation, fundoscopic examination was normal, but optical coherence tomography (OCT) performed when symptoms were present showed bilateral subretinal fluid (SRF) collections under the fovea (Figure 4). Toxicities in all subjects resolved promptly with the reduction or discontinuation of opaganib.

## 4 Discussion

This phase II study investigated the safety and efficacy of opaganib, an oral sphingolipid metabolism inhibitor, in combination with two of the most utilized NHAs in advanced prostate cancer, abiraterone and enzalutamide after progression on the NHA. Specifically, the study was intended to detect whether the addition of opaganib while continuing the original NHA could recapture response following progression of their disease on NHA. The study did not demonstrate disease control after 16 weeks of treatment in at least 30% of patients, with only 15% of patients in the abiraterone cohort and 9% of patients in the enzalutamide cohort experiencing disease control. Although the study was negative with respect to the primary endpoint, there was evidence of clinical activity in a subset of patients who were able to recapture a transient PSA response as indicated by a PSA30 of 23% and 6% in the abiraterone and enzalutamide cohorts, respectively. Opaganib, in combination with an NHA, was associated with a safety profile consistent with that seen in the phase I first‐in‐human trial of opaganib [20], and safety experience with abiraterone or enzalutamide monotherapy, respectively.

Opaganib, as a potential partner to an NHA, offered the prospect of maintaining a chemotherapy‐free period in CRCP, reflecting a patient‐centric approach that prioritizes the ability to remain on oral anticancer therapies whenever possible [19]. The potential for triplet therapy (docetaxel, an NHA, and androgen deprivation therapy [ADT]) exists in the first line setting for mCSPC after ARASENS and PEACE‐1 showed a benefit over docetaxel and ADT alone in those with high‐volume disease. However, the majority of patients will not receive chemotherapy until progression to mCRPC [27, 28, 29]. Many patients are treated upfront with hormonal doublet therapy, including an NHA and ADT [30]. There are patient‐reported outcome (PRO) data suggesting that quality of life decreases with the addition of chemotherapy relative to hormonal therapy [31]. In addition to the existing PRO data there are the common beliefs that chemotherapy is toxic, causes death in cancer patients, and should be avoided. These beliefs are supported by the well‐documented decrease in quality and length of life following administration of chemotherapy to subjects with poor performance status [32, 33]. Furthermore, some degree of toxicity over time is mitigated when visits to the infusion center are avoided with orally administered medications [34]. Many novel therapeutics garnering interest in patients with heavily pretreated CRPC over the last 2 years require isolation from family members and caregivers for several days after each administration, or prolonged time in a hospital or infusion center for observation after each dose [5, 35, 36].

Beyond being an oral agent, opaganib is a mechanistically intriguing partner as well. Through its in vitro capability to reduce AR expression in prostate cancer cells, there exists a rationale for opaganib rescue after secondary resistance develops against NHA and androgen blockade [16, 17, 18]. This study identified evidence of clinical activity in a subset of patients during a critical, pre‐chemotherapy point in the CRPC continuum. This type of trial design, namely the addition of a secondary agent like opaganib to recapture response to the original NHA, stands out among a background of recent trial designs in CRPC that utilize an experimental therapy plus a second line NHA after progression on the alternate NHA [5, 37]. The higher bar for efficacy in an “addition” versus a “switch” trial design may have put a statistically significant primary endpoint out of reach for opaganib plus abiraterone or enzalutamide. Given the negative primary efficacy endpoints from the current study, pursuing a Phase III trial that tests adding opaganib to NHA in CRPC, as done in the Phase II trial, is not planned. Subsequent therapeutic explorations of opaganib in CRPC should build on a biomarker approach. This work is ongoing and will be based on translational evidence of a prognostic role of circulating sphingolipids in NHA resistance among patients with metastatic prostate cancer [38].

Opaganib was, for the most part, safe and tolerable. As in the initial phase 1 trial of single‐agent opaganib [20], common treatment‐related toxicities were mild nausea and mild–moderate fatigue [20]. Adverse events of special interest experienced across cohorts in this trial and the previous phase I trial were treatment‐related symptoms classified as nervous system disorders. These included dizziness, dysarthria, dysgeusia, dysesthesias, headache, memory loss, muscle spasms, tremors, paresthesia, and somnolence [20, 39]. Some psychiatric disorders, including agitation/anxiety, mood changes, and hallucinations (both auditory and visual), were also experienced. Visual symptoms may now be attributable, in part, to direct ocular toxicity. We documented the accumulation of subretinal fluid that resolved with opaganib discontinuation. Subretinal fluid accumulation (central serous chorioretinopathy) is increasingly being recognized as a toxicity from anti‐cancer therapy. Implicated agents include kinase inhibitors targeting MEK, BRAF, FGFR2, as well as anti‐PD1 immunotherapy [40, 41, 42, 43, 44, 45, 46, 47]. Opaganib may be another kinase inhibitor capable of causing this toxicity. Future studies of this agent, as monotherapy or in combination with agents like checkpoint inhibitors (based on existing murine data supporting synergy), may benefit from the prospective use of OCT to monitor and prevent irreversible serous retinopathy [48].

This trial has several limitations to be acknowledged. This is ultimately a small sample size drawn from two academic medical centers, which may impact the generalizability of the results. The primary endpoint of the rate of disease control at week 16 (Day 113) was clinically meaningful but may have failed to capture patients who had a transient response to opaganib, or patients who stopped treatment early due to toxicity. Very few patients had measurable disease at baseline, and thus, RECIST measurements were available for a minority of patients; objective response rates were not calculable in this study for this reason. The trial design utilizing opaganib addition to each subject's NHA prior to study entry did not allow for assessment of the respective contribution of each component of the combination therapy. The contextual interpretation of the results is affected by the significant changes in the treatment landscape of prostate cancer by the time of the trial publication with the advent of widespread uptake of PET PSMA imaging, introduction of triplet therapy (docetaxel + NHA + ADT) in castration‐sensitive prostate cancer, and the FDA approval of radioligand therapies.

## 5 Conclusions

Sphingosine kinase 2 (SphK2) inhibition by opaganib works through multifactorial mechanisms to produce an anti‐cancer effect. Its ability to reduce AR expression in prostate cancer cells is of interest in emerging CRPC. This phase II study of opaganib in combination with abiraterone or enzalutamide showed evidence of clinical activity in a subset of patients but failed to meet its primary endpoint of disease control at Day 113 of treatment. As in the initial phase I trial, opaganib was generally well tolerated but also appeared specifically linked to uncommon visual and neuropsychiatric symptoms; there did not appear to be an amplification of toxicity in combination with NHA therapy. Subjects who experienced a PSA response or stabilization of their disease after the addition of opaganib to their NHA regimen warrant further genomic and biomarker exploration to determine which subsets of patients with prostate cancer may respond most favorably to this novel therapeutic approach, and we plan to present these data in a future report.

## Author Contributions

**Jacqueline T. Brown:** formal analysis (equal), investigation (equal), writing – original draft (lead), writing – review and editing (lead). **Bassel Nazha:** formal analysis (equal), investigation (equal), writing – original draft (lead), writing – review and editing (lead). **Anna C. Ferreira:** data curation (equal), formal analysis (equal), investigation (equal), writing – original draft (equal), writing – review and editing (equal). **Kent Armeson:** data curation (equal), formal analysis (equal), software (equal), supervision (equal), writing – original draft (equal). **Elizabeth G. Hill:** conceptualization (equal), data curation (equal), formal analysis (equal), methodology (lead), writing – original draft (equal), writing – review and editing (equal). **Besim Ogretmen:** writing – original draft (equal), writing – review and editing (equal). **Shikhar Mehrotra:** writing – original draft (equal), writing – review and editing (equal). **Alan Brisendine:** data curation (equal), formal analysis (equal), writing – original draft (equal), writing – review and editing (equal). **George Magrath:** data curation (equal). **Terry F. Plasse:** funding acquisition (equal), writing – original draft (equal), writing – review and editing (equal). **Theodore Stewart Gourdin:** writing – review and editing (equal). **Omer Kucuk:** conceptualization (equal), data curation (equal), writing – original draft (equal), writing – review and editing (equal). **Michael Lilly:** conceptualization (lead), data curation (equal), funding acquisition (equal), writing – original draft (equal), writing – review and editing (equal).

## Funding

This work was supported by grant P01 CA203628‐07A1 (B. Ogretmen, PI) from the National Cancer Institute, and by the Biostatistics Shared Resource, Hollings Cancer Center, Medical University of South Carolina (P30 CA138313). RedHill Biopharma generously provided clinical grade opaganib tablets.

## Disclosure

Translational Relevance: Opaganib is a first‐in‐class sphingolipid metabolism inhibitor with selective inhibitory activities on sphingosine kinase‐2 (SphK2) over SphK1, and on dihydroceramide desaturase (DES). It exhibits anticancer, anti‐inflammatory, and anti‐viral activities. In prostate cancer cell models, opaganib reduces androgen receptor expression, thus altering a primary resistance mechanism. This phase II trial investigated the safety and efficacy of opaganib in combination with abiraterone or enzalutamide in patients with castration‐resistant prostate cancer (CRPC). While the study did not meet its disease control efficacy endpoint, there was evidence of clinical activity in a subset of patients. Translational efforts are ongoing to determine the biomarker profile of patients who may respond most favorably to this therapeutic approach that aims to maintain a chemotherapy‐free period in CRPC.

## Conflicts of Interest

B.N. was a consultant or member of the advisory board for Exelixis, Intrinsic Specialty Solutions—AmerisourceBergen, Cardinal Health, Intellisphere, Neogenomics. He received institutional research funding from Xencor, Merck, Astellas, Seagen/Pfizer, and Exelixis. J.B. was a consultant or member of the advisory board for Exelixis, Gilead, and Xencor. She received institutional research funding from Medicenna, Surface Oncology, Xencor, Merck and Hookipa Pharma. M.L. is a consultant or collaborator for Senex Biopharma and Lipoimmunomedics. T.P is a consultant to RedHill Biopharma Ltd.

## Supporting information


**Data S1:** cam471633‐sup‐0001‐Supinfo.docx.

## Data Availability

Research data are not shared due to ethical considerations and privacy of the participating subjects. The research findings are summarized in the manuscript.
