# Supplementary material for: Phase II Trial of Opaganib Addition in Metastatic Castration‐Resistant Prostate Cancer After Disease Progression on Abiraterone or Enzalutamide
Source: Cancer Med. 2026 Apr 14;15(4):e71633. doi: 10.1002/cam4.71633 (PMC13079437; doi:10.1002/cam4.71633)
Supplement: Supplementary file 1 — Data S1: cam471633‐sup‐0001‐Supinfo.docx. [file CAM4-15-e71633-s001.docx]

**SUPPLEMENTAL MATERIAL**

**Supplemental Table 1**. Frequency of All Adverse Events by Grade within System Organ Class (SOC) for All Cohorts

| **SOC**, n (%)  Adverse Event, n (%) | All Cohorts N = 66 | | | | |
| --- | --- | --- | --- | --- | --- |
|  | Grade | | | | |
|  | 1 | 2 | 3 | 4 | 5 |
| **Blood** | **1 (2)** | **1 (2)** | **6 (9)** | **—** | **—** |
| Anemia | 1 (2) | 1 (2) | 6 (9) | — | — |
| Leukocytosis | 1 (2) | — | 1 (2) | — | — |
| **Cardiac** | **1 (2)** | **—** | **1 (2)** | **—** | **2 (3)** |
| Myocardial Infarction | — | — | — | — | 1 (2) |
| Palpitations | 1 (2) | — | — | — | — |
| Sinus Tachycardia | — | — | 1 (2) | — | — |
| Ventricular Arrhythmia | — | — | — | — | 1 (2) |
| **Ear** | **3 (5)** | **—** | **—** | **—** | **—** |
| Tinnitus | 2 (3) | — | — | — | — |
| Vertigo | 1 (2) | — | — | — | — |
| **Eye** | **6 (9)** | **1 (2)** | **—** | **—** | **—** |
| Blurred Vision | 2 (3) | 1 (2) | — | — | — |
| Flashing Lights | 3 (5) | — | — | — | — |
| Floaters | 1 (2) | — | — | — | — |
| **Gastrointestinal** | **24 (36)** | **4 (6)** | **1 (2)** | **—** | **—** |
| Abdominal Distension | 1 (2) | — | — | — | — |
| Abdominal Pain | 5 (8) | — | 1 (2) | — | — |
| Bloating | 1 (2) | — | — | — | — |
| Constipation | 7 (11) | 1 (2) | — | — | — |
| Diarrhea | 6 (9) | — | — | — | — |
| Dry Mouth | 3 (5) | — | — | — | — |
| Dyspepsia | 2 (3) | — | — | — | — |
| Dysphagia | 1 (2) | 1 (2) | — | — | — |
| Fecal Incontinence | — | 2 (3) | — | — | — |
| Flatulence | 1 (2) | — | — | — | — |
| Gastroesophageal Reflux Disease | 2 (3) | — | — | — | — |
| Nausea | 11 (17) | — | — | — | — |
| Oral Dysesthesia | — | 1 (2) | — | — | — |
| Vomiting | 3 (5) | — | — | — | — |
| **General** | **22 (33)** | **7 (11)** | **2 (3)** | **—** | **—** |
| Chills | 1 (2) | — | — | — | — |
| Edema Limbs | 3 (5) | 1 (2) | — | — | — |
| Fatigue | 16 (24) | 4 (6) | 1 (2) | — | — |
| Fever | 2 (3) | 2 (3) | — | — | — |
| Gait Disturbance | 1 (2) | — | — | — | — |
| Injection Site Reaction | 1 (2) | — | — | — | — |
| Localized Edema | 1 (2) | — | — | — | — |
| Malaise | 1 (2) | — | — | — | — |
| Pain | 3 (5) | — | 1 (2) | — | — |
| **Infections** | **4 (6)** | **1 (2)** | **—** | **2 (3)** | **—** |
| Catheter Related Infection | 1 (2) | — | — | — | — |
| Sepsis | — | 1 (2) | — | 2 (3) | — |
| Sinusitis | 1 (2) | — | — | — | — |
| Tooth Infection | 1 (2) | — | — | — | — |
| Upper Respiratory Infection | 3 (5) | — | — | — | — |
| Urinary Tract Infection | — | 1 (2) | 1 (2) | — | — |
| **Injury** | **5 (8)** | **1 (2)** | **—** | **—** | **—** |
| Bruising | 2 (3) | — | — | — | — |
| Fall | 1 (2) | 1 (2) | — | — | — |
| Fracture | 2 (3) | — | — | — | — |
| **Investigations** | **4 (6)** | **3 (5)** | **3 (5)** | **1 (2)** | **—** |
| Activated Partial Thromboplastin Time Prolonged | 1 (2) | 2 (3) | — | — | — |
| Alkaline Phosphatase Increased | 1 (2) | — | 1 (2) | — | — |
| Blood Bilirubin Increased | 1 (2) | — | 1 (2) | — | — |
| Creatinine Increased | — | 1 (2) | 1 (2) | — | — |
| Lymphocyte Count Decreased | — | — | 1 (2) | — | — |
| Platelet Count Decreased | — | — | — | 1 (2) | — |
| Weight Gain | 1 (2) | — | — | — | — |
| Weight Loss | 1 (2) | — | — | — | — |
| **Metabolism** | **8 (12)** | **2 (3)** | **1 (2)** | **1 (2)** | **—** |
| Anorexia | 8 (12) | — | — | — | — |
| Dehydration | — | 2 (3) | 1 (2) | — | — |
| Hyperkalemia | 1 (2) | — | — | — | — |
| Hypokalemia | 3 (5) | — | — | — | — |
| Hypomagnesemia | 1 (2) | — | — | — | — |
| Hyponatremia | — | — | — | 1 (2) | — |
| **Musculoskeletal** | **11 (17)** | **4 (6)** | **4 (6)** | **—** | **—** |
| Arthralgia | 2 (3) | — | — | — | — |
| Arthritis | — | 1 (2) | — | — | — |
| Back Pain | 4 (6) | — | 3 (5) | — | — |
| Bone Pain | 4 (6) | — | — | — | — |
| Chest Wall Pain | 1 (2) | — | — | — | — |
| Flank Pain | 1 (2) | 1 (2) | 1 (2) | — | — |
| Generalized Muscle Weakness | 2 (3) | 1 (2) | — | — | — |
| Muscle Cramp | 1 (2) | — | — | — | — |
| Muscle Weakness Lower Limb | 2 (3) | — | — | — | — |
| Muscle Weakness Right sided | 1 (2) | — | — | — | — |
| Neck Pain | 1 (2) | — | 1 (2) | — | — |
| Pain In Extremity | 1 (2) | 2 (3) | 1 (2) | — | — |
| **Nervous** | **20 (30)** | **8 (12)** | **1 (2)** | **1 (2)** | **—** |
| Ataxia | 1 (2) | — | — | — | — |
| Depressed Level Of Consciousness | 1 (2) | — | — | — | — |
| Dizziness | 11 (17) | 2 (3) | — | — | — |
| Dysarthria | 1 (2) | — | — | — | — |
| Dysgeusia | 3 (5) | 2 (3) | — | — | — |
| Edema Cerebral | — | — | — | 1 (2) | — |
| Headache | 7 (11) | — | — | — | — |
| Intracranial Hemorrhage | — | 1 (2) | — | — | — |
| Lethargy | — | 1 (2) | — | — | — |
| Memory Impairment | 1 (2) | 1 (2) | — | — | — |
| Paresthesia | 1 (2) | — | — | — | — |
| Peripheral Motor Neuropathy | — | 1 (2) | — | — | — |
| Peripheral Sensory Neuropathy | 3 (5) | — | — | — | — |
| Seizure | — | 1 (2) | — | — | — |
| Stroke | — | — | 1 (2) | — | — |
| Transient Ischemic Attacks | 1 (2) | — | — | — | — |
| Tremor | 1 (2) | — | — | — | — |
| **Psychiatric** | **5 (8)** | **4 (6)** | **—** | **—** | **—** |
| Anxiety | — | 1 (2) | — | — | — |
| Confusion | 3 (5) | 2 (3) | — | — | — |
| Depression | 1 (2) | — | — | — | — |
| Hallucinations | 3 (5) | 1 (2) | — | — | — |
| Restlessness | 1 (2) | — | — | — | — |
| **Renal** | **6 (9)** | **4 (6)** | **—** | **—** | **—** |
| Bladder Spasm | 1 (2) | — | — | — | — |
| Hematuria | 2 (3) | 1 (2) | — | — | — |
| Proteinuria | — | 2 (3) | — | — | — |
| Urinary Frequency | 1 (2) | — | — | — | — |
| Urinary Incontinence | 2 (3) | 1 (2) | — | — | — |
| Urinary Tract Obstruction | 1 (2) | 1 (2) | — | — | — |
| Urinary Tract Pain | 1 (2) | — | — | — | — |
| **Reproductive** | **2 (3)** | **1 (2)** | **—** | **—** | **—** |
| Pelvic Pain | 2 (3) | 1 (2) | — | — | — |
| **Respiratory** | **3 (5)** | **—** | **1 (2)** | **—** | **—** |
| Dyspnea | 3 (5) | — | — | — | — |
| Pleural Effusion | 1 (2) | — | — | — | — |
| Pneumonitis | — | — | 1 (2) | — | — |
| Sinus Disorder | 1 (2) | — | — | — | — |
| **Skin** | **6 (9)** | **—** | **—** | **—** | **—** |
| Hyperhidrosis | 1 (2) | — | — | — | — |
| Pruritus | 1 (2) | — | — | — | — |
| Rash Acneiform | 3 (5) | — | — | — | — |
| Skin Atrophy | 1 (2) | — | — | — | — |
| **Vascular** | **6 (9)** | **4 (6)** | **2 (3)** | **—** | **—** |
| Flushing | 1 (2) | — | — | — | — |
| Hematoma | — | 1 (2) | — | — | — |
| Hot Flashes | 4 (6) | — | — | — | — |
| Hypertension | 1 (2) | 2 (3) | 2 (3) | — | — |
| Hypotension | 1 (2) | — | — | — | — |
| Thromboembolic Event | 1 (2) | 1 (2) | — | — | — |

**Supplemental text: Full list of inclusion and exclusion criteria**

### **Inclusion Criteria**

- Patient must have mCRPC. Each patient must have: * Tissue diagnosis documented by pathology report, or clinic note attesting to same * Radiographically-demonstrated metastases * Patients must have adenocarcinoma, or ductal carcinoma, or combinations of these two entities
- Voluntary, signed and dated, Institutional Review Board (IRB)-approved informed consent form in accordance with regulatory and institutional guidelines
- Documented progression during treatment with enzalutamide or abiraterone, as determined by the enrolling investigator
- Testosterone level documented to be less than 50 ng/dL
- Eastern Cooperative Oncology Group (ECOG) performance status of 0-2
- Bilirubin =< 1.5 times upper limit of normal (Common Terminology Criteria for Adverse Events [CTCAE] grade 1 baseline)
- Aspartate aminotransferase (AST) (serum glutamic oxaloacetic transaminase [SGOT]) & alanine aminotransferase (ALT) (serum glutamic pyruvic transaminase [SGPT]) =< 3 x upper limit of normal (ULN) (CTCAE grade 1 baseline)
- Subjects with Gilbert’s syndrome may be included if the total bilirubin is < 3 x ULN and the direct bilirubin is within normal limits
- Serum creatinine =< 1.5 x ULN (CTCAE grade 1 baseline)
- Absolute neutrophil count >= 1000 cells/mm^3
- Platelet count >= 75,000 (plt/mm^3) (CTCAE grade 1 baseline)
- Hemoglobin >= 9.0 g/dL
- Fasting blood glucose of < 165 mg/dL or random blood glucose of < 200 mg/dL
- Urinalysis: no clinically significant abnormalities
- International normalized ratio (INR) =< 1.7 for patients not on anti-coagulation medications (meds)
- Well-controlled blood pressure as determined by the treating investigator
- Patients requiring narcotic analgesics must be on stable doses for at least 2 weeks prior to study entry

### **Exclusion Criteria**

- New York Heart Association class III or IV, cardiac disease, myocardial infarction within the past 6 months, unstable arrhythmia, or evidence of ischemia on electrocardiogram (ECG)
- Underlying psychiatric disorder requiring hospitalization within the last two years
- Clinically significant neurological disorder (Parkinson’s disease, dementia, multiple sclerosis), as determined by the enrolling investigator
- Active, uncontrolled bacterial, viral or fungal infection, requiring systemic therapy
- Treatment with radiation therapy, surgery, or investigational therapy within 28 days prior to registration
- Unwillingness or inability to comply with procedures required in this protocol
- Serious nonmalignant disease that could compromise protocol objectives in the opinion of the investigator
- Patients who are receiving coumadin, apixaban, or rivaroxaban. Patients who are receiving other drugs that are sensitive substrates of CYP450 1A2, 3A4, 2B6, 2C8, 2C9, 2C19 or 2D6, P-gP, BCRP, and OATP1B1, or strong inhibitors or inducers of all major CYP450 isozymes that cannot be stopped at least 7 days or 5 half-lives (whichever is longer) before starting treatment with opaganib may be treated on this study with careful monitoring for toxic effects or loss of efficacy of the relevant drug
- Patients who are currently participating in any other clinical trial of an investigational product
- Other primary malignancy requiring systemic treatment within past 5 years except carcinoma in situ of the cervix or urinary bladder or non-melanoma skin cancer
- Any other mental incapacitation or psychiatric illness that would preclude study participation, as determined by the enrolling investigator
- Prisoners or patients who are compulsorily detained (involuntarily incarcerated) for treatment of either a psychiatric or physical (e.g., infectious disease) illness must not be enrolled into this study
- Patients that have had chemotherapy for castration resistant prostate cancer (patients can have had chemotherapy for castration sensitive PC) * Exception: Patients who had prior chemo for CRPC are eligible if they have a performance status (PS) = 0-1 and life expectancy of more than 6 months
